# Wild Isolates of *Neurospora crassa* Reveal Three Conidiophore Architectural Phenotypes

**DOI:** 10.3390/microorganisms8111760

**Published:** 2020-11-09

**Authors:** Emily K. Krach, Yue Wu, Michael Skaro, Leidong Mao, Jonathan Arnold

**Affiliations:** 1 Genetics Department, University of Georgia, Athens, GA 30602, USA; ekrach@uga.edu; 2 Institute of Bioinformatics, University of Georgia, Athens, GA 30602, USA; yue.wu@uga.edu (Y.W.); Michael.skaro@uga.edu (M.S.); 3 School of Electrical and Computer Engineering, College of Engineering, University of Georgia, Athens, GA 30602, USA; mao@uga.edu

**Keywords:** *Neurospora crassa*, natural variation, complex trait, conidiophore development, phenomics, convolutional neural network, generalized linear model, spore shadow

## Abstract

The vegetative life cycle in the model filamentous fungus, *Neurospora crassa,* relies on the development of conidiophores to produce new spores. Environmental, temporal, and genetic components of conidiophore development have been well characterized; however, little is known about their morphological variation. We explored conidiophore architectural variation in a natural population using a wild population collection of 21 strains from Louisiana, United States of America (USA). Our work reveals three novel architectural phenotypes, Wild Type, Bulky, and Wrap, and shows their maintenance throughout the duration of conidiophore development. Furthermore, we present a novel image-classifier using a convolutional neural network specifically developed to assign conidiophore architectural phenotypes in a high-throughput manner. To estimate an inheritance model for this discrete complex trait, crosses between strains of each phenotype were conducted, and conidiophores of subsequent progeny were characterized using the trained classifier. Our model suggests that conidiophore architecture is controlled by at least two genes and has a heritability of 0.23. Additionally, we quantified the number of conidia produced by each conidiophore type and their dispersion distance, suggesting that conidiophore architectural phenotype may impact *N. crassa* colonization capacity.

## 1. Introduction

*Neurospora crassa* propagates asexually through the dissemination of haploid spores, conidia, which develop via specialized aerial structures called conidiophores [1]. Macroconidiophores give rise to macroconidia and are morphologically and developmentally distinct from their smaller counterparts, microconidiophores, which give rise to uninuclear microconidia [2]. Since macroconidiophores are the subject of this work, they are hereafter referred to simply as conidiophores. Development of conidiophores is under strict environmental and temporal control, requiring cues such as desiccation, nutrient deprivation, and light exposure for its induction [3]. After exposure to these environmental triggers, aerial hyphae grow perpendicular to the preceding mycelial mat to stimulate conidiophore development. The organism subsequently undergoes a series of constriction budding followed by crosswall formations until eventual sporulation, roughly 10 hours (h) following the beginning of this process [4]. It is through this dissemination of conidia that the vegetative life cycle can propagate, thus allowing new filaments to germinate after a period of dormancy. Conidiophore development is under strict circadian control, requiring activation of the white collar complex (WCC) by light for activation of *fluffy* (*fl*), a necessary regulator of conidiophore development [5,6]. While environmental, temporal, and genetic components of conidiophore development in *N. crassa* have been well characterized [7], little is known about morphological variation of these structures, particularly in natural populations.

In other filamentous fungal species, conidiophore architecture has been shown to directly impact the ability of an organism to disseminate throughout an environment and infect host tissues. For example, conidiophore morphology is altered in *Aspergillus niger* following deletion of *velvetA*, directly impacting both spore dispersion and colonization capacity of the organism [8]. Additionally, mutating the *acropetal* locus of *Magnaporthe grisea* modifies arrangement of the developing spores in a conidiophore, resulting in spores that are nonpathogenic [9]. Given these known effects in other fungal species, we sought to characterize natural conidiophore architectural variation in *N. crassa*, estimate heritability of these phenotypes, and examine their potential impact on sporulation. To do this, we employed a wild population collection of 21 *N. crassa* strains collected from Louisiana, United States of America (USA) [10]. These isolates have been used in previous population genetics studies to elucidate local adaptations to cold tolerance and circadian rhythm [11] and characterize novel functions of loci [12,13]. Our work reveals three novel and distinct conidiophore architectural phenotypes among these wild populations. These phenotypes persist throughout the duration of conidiophore development and are described here as Wild Type (WT), Bulky, and Wrap. By conducting crosses between each phenotype and characterizing subsequent progeny conidiophores, we were able to fit a model for heritability of the discrete complex trait, conidiophore type. Our model suggests that at least two genes control conidiophore architectural phenotype and estimates a heritability of 0.23. We explored the potential impact of conidiophore phenotype on sporulation by quantifying the number of conidia produced and their dispersal distance. Furthermore, we present a novel approach with an accurate image-classifier using a convolutional neural network [14] specifically developed to assign conidiophore architectural phenotypes in a high-throughput manner.

## 2. Materials and Methods

### 2.1. Strains and Media

Wild Louisiana isolates were obtained from the Fungal Genetics Stock Center (FGSC, Manhattan, KS, USA) [15] and are listed in Table A1 (Appendix A). Strains were maintained on 1.8% glucose/1.8% fructose/1.5% agar slants with 1× Vogel’s media and recommended biotin and trace element supplements. To isolate conidiophores, strains were inoculated on 1.8% glucose/1.8% fructose/1.5% agar plates (100 × 15 millimeters (mm)) with 1× Vogel’s media, biotin, and trace element supplements and incubated at 30 °C for 20 h. Cultures were then harvested onto a 82 mm diameter nitrocellulose membrane with 0.45 micron (µm) pore size (Whatman Protran BA-85, Maidstone, England), inverted onto a new agar plate as described above, and placed under light for aerial hyphae to penetrate the membrane. After 20 h, membranes were removed from the agar and secured on a flat surface for imaging of conidiophores. Method was adapted from Bailey-Shrode and Ebbole, 2004 [6].

Cultures for time-course images were grown following the same protocol with nitrocellulose membranes removed at 20, 25, or 35 h for imaging of conidiophores at different stages of development.

### 2.2. Crosses and Progeny Screening

Crosses were performed in the dark on cornmeal crossing medium, after which ascospores were plated on sorbose + fructose + glucose (SFG) media. Colonies were subsequently picked to isolate random ascospore progeny. Strains selected for crossing were FGSC8872 (WT) × FGSC3943 (Bulky), FGSC8872 (WT) × FGSC8876 (Wrap), and FGSC2229 (Bulky) × FGSC8876 (Wrap). Crosses were conducted in duplicate with 25 progeny selected from each. To isolate and image conidiophores in a high-throughput manner while preventing fusion of different progeny sharing a plate, each strain was inoculated on a 1 mL 1.5% agar droplet containing 1.8% glucose/1.8% fructose with 1× Vogel’s media and recommended biotin and trace element supplements. Each 150 × 15 mm petri dish contained eight agar droplets evenly spaced roughly 2.5 cm apart. Each droplet was inoculated with progeny conidia and incubated at 30 °C for 20 h to allow sufficient mycelial growth without hyphal fusion between droplets. Each droplet was then harvested onto a separate nitrocellulose membrane with 0.45 µm pore size. Each membrane was inverted onto a new agar droplet as described above and placed under light for aerial hyphae to penetrate the membrane. After 25 h, membranes were removed from the agar and secured on a flat surface for imaging of conidiophores.

### 2.3. Microscopy and Image Deconvolution

Nitrocellulose membranes containing conidiophores were visualized on an inverted microscope (Axio Observer A1, Carl Zeiss Microscopy, LLC, Thornwood, NY, USA) at 20× magnification and brightfield images were taken with a charge-coupled device (CCD) camera (AxioCam HRm, Carl Zeiss Microscopy, LLC, Thornwood, NY, USA). Multiple z-slices were captured and overlaid in ImageJ [16] to convey a complete representation of each three-dimensional structure. Augmentation including contrast enhancement and noise and background subtraction was conducted on image stacks to isolate conidiophores from underlying mycelia and/or aerial hyphae [17].

### 2.4. Automated Phenotype Classification

Residual nets (ResNet) was used to classify brightfield images into the three phenotypic classes by transfer learning [14]. After some hyperparameter searching, ResNet-50 pretrained on ImageNet was chosen as the starting neural network for image classification [18]. Training parameters were as follows: batch size, 15; initial learning rate, 0.001; stochastic gradient decent as the optimizer. The training process did not update parameters before module 3 in ResNet, and the parameters were only updated for the last 27 convolutional layers and the fully connected layer. Learning rates decayed when plateau was used for modifying learning. Augmentation including random rotation, random horizontal flip, and random gray was introduced in the training to simulate realistic diversity and prevent overfitting. Before augmentation, all images went through padding and resize to fit the ResNet input size. All images were converted to tensor and normalized.

**Figure A1:**
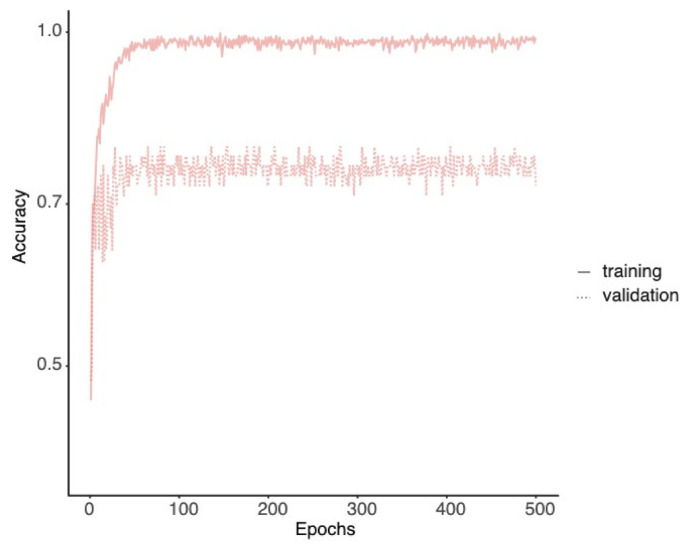
Model accuracies through training. Accuracies of the training (solid line) and validation (dotted line) sets is plotted through epochs. The final model was chosen on the basis of the accuracy of the validation set.

We balanced the three classes of conidiophore phenotypes and separated the dataset into training (543), validation (66), and test sets (66). An external test set generated from progeny conidiophore images was collected in a separate batch and used for further validation (WT: 40; Bulky: 60; Wrap: 49). The training set was used to train the neural network and update its parameters. The validation set was used for updating learning rates and finding the final model to alleviate overfitting (Figure A1 in the Appendix A). The test set was not used until the final performance evaluation. We calculated accuracy and macro definition of precision and recall for multiclass classification [19]. The model training and inference used PyTorch [20], torchvision, and CUDA. P100 at Sapelo2 of GACRC was used for model training.

Feature importance was also evaluated on the test set. Features were evaluated on an integrated gradient [21], and the noise tunnel method [22] smoothed the result. We chose the maximal value as baseline instead of regular choices (constant black) because, in our brightfield images, a lighter background indicated the absence of features [23]. In noise tunnel, the noise was added 10 times, and the means of squared attributions were used.

The codes for training and visualization are shared on GitHub (https://github.com/michaelSkaro/image_classification/tree/master/src).

### 2.5. Sporulation Quantification

Representative strains for each phenotypic class (FGSC8872, FGSC8876, and FGSC3943) were inoculated on 1.8% glucose/1.8% fructose/1.5% agar plates with 1× Vogel’s media, biotin, and trace element supplements and incubated at 30 °C for 30 h. Three biological replicates of each strain were performed. Cultures were then harvested onto an 82 mm diameter nitrocellulose membrane and inverted onto a new agar plate as previously described above. Plates were placed under light for 32 h to allow penetration of aerial hyphae, development of conidiophores, and subsequent sporulation. Nitrocellulose membranes were then removed, and conidia were suspended into 50 mL of water and counted and sized with the Cellometer Auto 2000 (Nexcelom, Inc., Lawrence, MA, USA) [24,25].

To compare dispersal distance traveled by spores of each phenotype, the same representative strains listed above were inoculated onto the same growth medium. After 20 h at 30 °C, cultures were harvested onto a 60 mm diameter nitrocellulose membrane, inverted onto a new plate, and set under light for 30 h. Membranes were then removed and placed at the center of a 245 mm square bioassay dish, where SFG medium surrounded a 60 mm diameter blank space now occupied by the membrane. The dish was placed under light for 48 h to allow for sporulation and subsequent colonial growth. At this time, pictures of each plate were taken with an iPhone XS, and contrast enhancement was performed in ImageJ. ImageJ was used to measure distance from the center of each nitrocellulose membrane to the center of each colony. Two biological replicates were conducted for each strain.

## 3. Results

### 3.1. Wild N. crassa Isolates Exhibit Three Conidiophore Architectural Phenotypes

**Figure 1:**
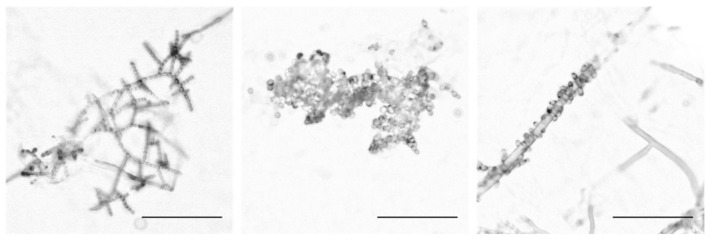
The three conidiophore architectural phenotypes. The Wild-Type (WT) phenotype is depicted in the first panel with FGSC2489, followed by the Bulky phenotype with FGSC8878, and Wrap with FGSC8876. Scale bar, 100 microns (µm).

Conidiophores from 21 wild Louisiana populations of *N. crassa* (Table A1) were isolated and imaged, and we identified striking architectural variation both within and between populations. Conidiophores were classified by their morphology into three groups that are hereafter referred to as Wild Type (WT), Bulky, and Wrap. The WT phenotypic class is characterized by linear chains of developing conidia that extend and branch outward from the aerial hypha. Developing spores in Bulky conidiophores, however, form crowded clusters that inhibit the ability to distinguish linear chains. These spores also display more variation in their size and shape. In the Wrap phenotype, conidia cling to and/or wrap around a hyphal filament rather than extending and branching outward as in the other phenotypic groups (Figure 1).

Each brightfield image was manually classified into one (or two) of the phenotypic classes using the guidelines described above (Table 1). Many strains had one phenotype demonstrating a clear majority, although every population had representation from at least two of the three phenotypes. All strains developed conidiophores with WT and Wrap phenotypes. Most strains also had Bulky conidiophores, with the exception of five populations (FGSC0847, FGSC2222, FGSC2228, FGSC3199, FGSC8872) lacking such individuals. Among the population set of 21 wild strains, 13 showed a majority of WT conidiophores, while five had a majority of Bulky, and three had a majority of Wrap.

### 3.2. Architectural Phenotypes Are Consistent throughout Conidiophore Development

**Figure 2:**
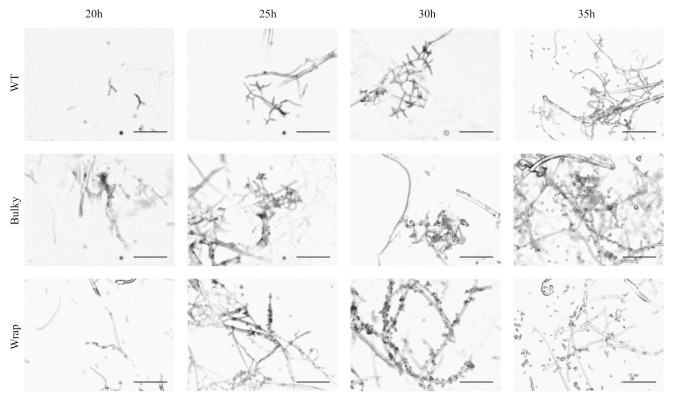
Development of conidiophore architectural phenotypes over time. Individual cultures were imaged at 20, 25, 30 and 35 h after being transferred to a nitrocellulose membrane and placed under light. Development of the conidiophore begins at roughly 20 h and concludes with sporulation by 35 h. Representative strains used were as follows: WT, FGSC2489; Bulky, FGSC8879; Wrap, FGSC8876. Scale bar, 100 µm.

To ensure the three phenotypes were not just representations of different time points along the same developmental trajectory, we captured images of strains strongly representing each phenotype at 20, 25, 30, and 35 h after transferring cultures to the nitrocellulose membrane, with 30 h as the initial reference point for mature conidiophore images. It is important to note that each time point was collected from a separate set of cultures, and these are not time-series images. WT, Bulky, and Wrap phenotypes were presented as early as 20 h and maintained until sporulation captured at 35 h, suggesting that the three architectural phenotypes are not a result of temporal disparity along the same developmental trajectory (Figure 2).

### 3.3. There Is No Dependence of Phenotype on Strain Collection Environment

**Figure 3:**
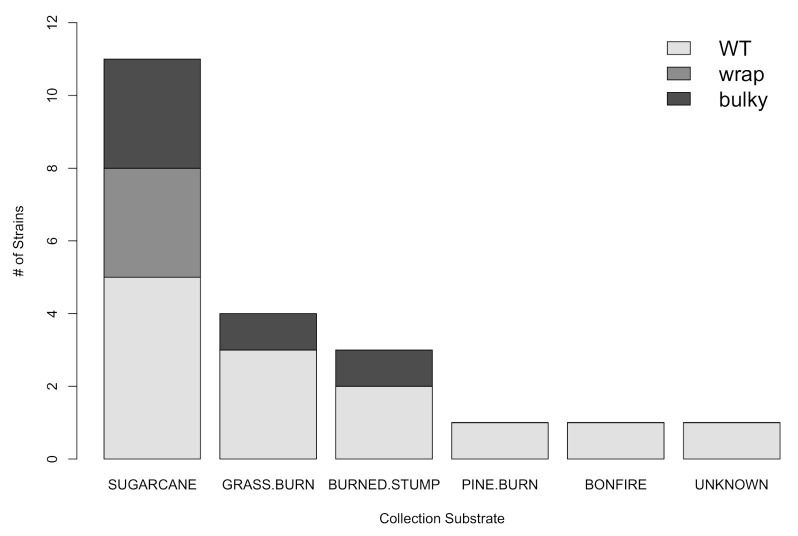
There is no significant correlation between the substrate from which a strain was collected and its most prominent phenotype (Χ^2^ = 4.9196, df = 10, *p* = 0.8965) from Table 1. Collection substrate as reported by the Fungal Genetics Stock Center (FGSC).

**Figure 4:**
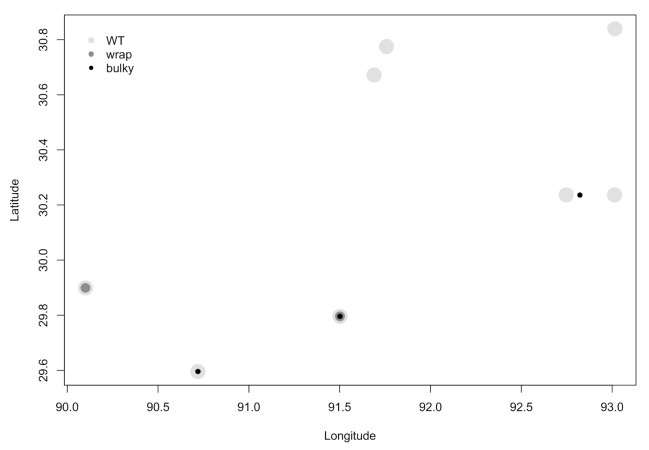
There is no clear relationship between most prominent phenotype and collection site. Louisiana town names were reported by the FGSC and are depicted here as geographical coordinates. Overlapping datapoints depict multiple strains collected from the same town.

Next, we investigated whether or not there was a correlation between the environment from which a strain was collected and its most prominent conidiophore phenotype. We found no statistically significant dependence of phenotype on collection substrate as reported by the Fungal Genetics Stock Center (FGSC) [15] (chi-squared test for independence; Χ^2^ = 4.9196, df = 10, *p* = 0.8965) (Figure 3). Interestingly, all strains with a majority of Wrap conidiophores (*n* = 3) were collected from sugarcane, although WT and Bulky strains were also found on this substrate. WT strains were found on all six substrates (sugarcane, grass burn, burned stump, pine burn, bonfire, and unknown), and Bulky strains were found on sugarcane, grass burn, and pine burn. We also did not find any clear relationship between most prominent phenotype and the town from which each strain was collected, as reported by FGSC (Figure 4).

### 3.4. Architectural Phenotypes Can Be Automatically Classified and Corresponding Features Can Be Extracted

Manually categorizing conidiophores into phenotypic classes is time-consuming and introduces potential bias. To streamline the future classification of additional conidiophore images, an automated classification process was developed. We demonstrated that an accurate image classifier could be constructed based on the limited conidiophore image dataset of 543 training samples and presents reasonable performances (Table 2 and Table 3).

**Figure 5:**
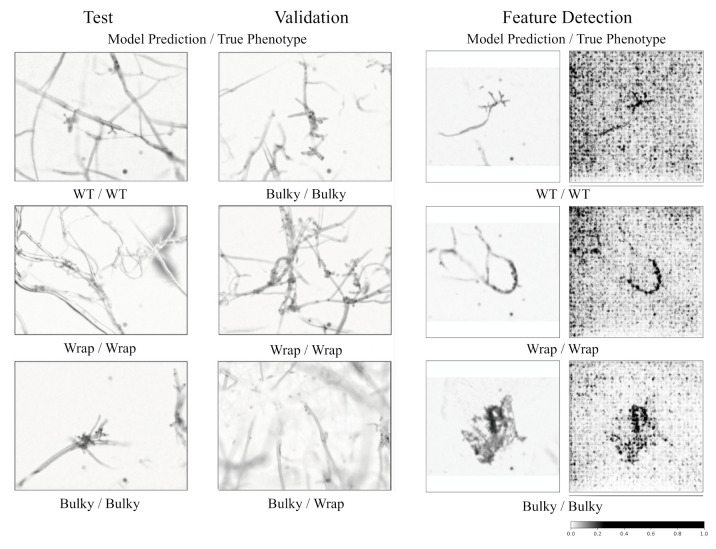
Automatic classification of phenotypes. Model performance on test and validation sets is depicted in the first two columns. The far-left column shows a selected test set, followed by an example validation set in the second column. The third and fourth columns show feature importance evaluation for a few test samples. The third column shows the original image (after padding and resize) with the last column identifying feature importance. A higher value (black) in the last column indicates greater feature importance. All images are labeled with the model predicted phenotype first and manually identified phenotype second.

Images from both the test and the validation set can be accurately classified at rates of 76% and 79%, respectively (Figure 5). Testing on an external dataset from a separate batch showed slightly worse performance at 68% accuracy. Feature importance is also evaluated (Figure 5), confirming that the program is properly identifying the conidiophore within an image for classification. This method was used to classify images of progeny conidiophores in a high-throughput manner to estimate potential heritability of these phenotypes, as described below.

### 3.5. Crosses Suggest at Least 2–3 Genes Involved in Conidiophore Architectural Phenotypes

Three crosses were performed between representative strains for each architectural phenotype: FGSC8872 × FGSC8876 (WT × Wrap), FGSC8876 × FGSC2229 (Wrap × Bulky), and FGSC 8872 × FGSC3943 (WT × Bulky). A total of 50 progeny were selected from each cross, from which conidiophores were isolated and imaged. For efficiency, these 1932 images were each assigned a phenotype by automatic classification as described above. These phenotype counts were then used to estimate an inheritance model for this discrete complex trait, described below (Table 4).

**Figure 6:**
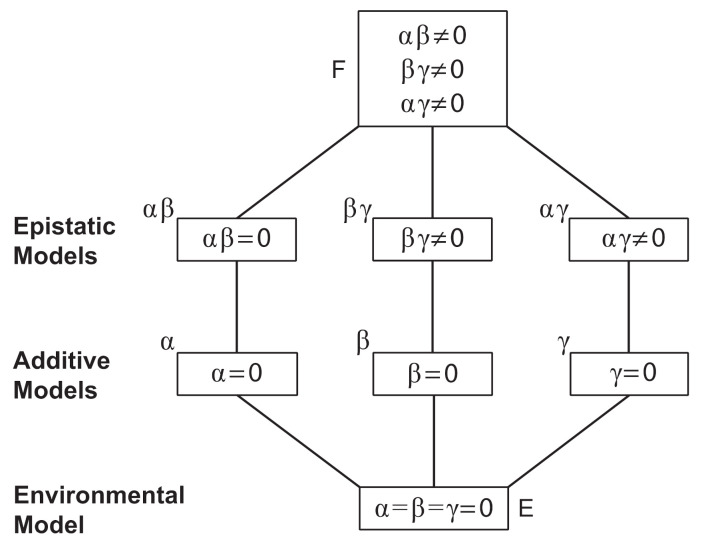
The above hierarchy of models contained particular models that fit the phenotypic counts on three crosses between parents with different conidiophore phenotypes. At the top of the model hierarchy is the full epistatic model (F) with a grand mean, three additive allelic effects, and three epistatic interactions. At the bottom of the hierarchy is an environmental model with no genetic effects on conidiophore phenotype (E). Intervening models in the hierarchy drop one or more parameters describing the additive and epistatic effects of genes A, B, and C associated with the conidiophore phenotypes, A, B, and C.

The inheritance model is summarized in the table above and motivated by classic models for quantitative traits [26]. In this model, each cross contributes the effect of one dominant allele at each of three loci. For example, in *Emericella (Aspergillus) nidulans*, at least three genes control conidiophore development [27]. The main effects of the three loci are denoted by α, β, and γ for the A (WT), B (Wrap), and C (Bulky) loci and associated phenotype, respectively. In each cross, there are two dominant alleles (one from each parent) being contributed that epistatically interact to determine the conidiophore architectural phenotype. In principle, these pairwise interactions could result from environmental interactions among progeny in the cross and/or developmental interactions within developing progeny. By isolating progeny in their own agar droplet (see Section 2), the former possibility could be eliminated. The pairwise interactions are denoted by αβ, βγ, and αγ. In addition, in this model, there is one grand mean λ that is adjusted so that all these probabilities of a progeny phenotype, K_ij_, sum to 1. The data tabled below are the multinomial counts of progeny from each of the three crosses (Table 5):

A denotes an allele associated with the WT phenotype, B denotes an allele associated with the Wrap phenotype, and C denotes an allele associated with the Bulky phenotype. These counts are stored in a 9 × 1 vector $\underline{N}$, the “dependent variable” to be explained by the allelic and epistatic effects. The sum of these nine progeny counts is *N*. Under this model, the log of the expectations of these counts E(Y) are linear in the parameters of the model, such as the allelic effects; thus, the model is sometimes referred to as a loglinear model [28]. Much as in a probit model, the goal of this discrete trait model is to associate an underlying quantitative trait through the log of the expected counts with the allelic and epistatic effects. Under the simplest hypothesis, all nine of these cell probabilities are equal. Departures from this hypothesis (H0) summarize the variation in the counts as measured by the Pearson $X_{0}^{2}$. Departures from the next simplest hypothesis (H1) summarize the additive variation in the counts as measured by the Pearson $X_{1}^{2}$. Departures from the last, most complicated hypothesis (H2) with all three interactions summarizes the additive and epistatic variation by the Pearson $X_{2}^{2}$. The heritability is summarized by the ratio of the additive variation to the total variation: H2 = ($X_{0}^{2}-X_{1}^{2})/X_{0}^{2}$. Many intervening models can be envisioned in a hierarchy of possible inheritance models (Figure 6).

Each model was fitted by the Method of Maximum Likelihood using iteratively reweighted least squares (IRLS) [29,30]. Under this approach, the information A about the counts is denoted by A, and the derivatives of the cell probabilities in the model above are stored in the array X tabled below (Table 6). The matrix NX’AX is the information about the parameters in the model. The score vector S represents the derivatives of the log of the cell probabilities and can be written as S = AY, where A is a 9 × 9 diagonal matrix where A(i,i) = 1/K(i,i).

The fitting of this model with seven parameters or a simplification of this inheritance model was obtained from solving the below weighted least squares problem where Y = S + NAX$\underline{N}$ is the provisional quantitative dependent variable, X is the regression matrix of independent variables, such as allelic effects, A is the weight matrix, and the parameters are $\beta^{\prime }=\left(\;\lambda ,\;\alpha ,\beta ,\gamma ,\alpha \beta ,\beta \gamma ,\alpha \gamma \right)$. The parameters are found by solving the following normal equation iteratively:

(1) $$NX^{\prime }AX\beta^{*}=\;X^{\prime }Y.$$

The matrix A and vector Y are evaluated with the current provisional parameter estimate β, and then the normal equations are solved for the updated parameter vector $\beta^{*}$. The process is repeated until the relative error is less than 10^−8^. The process is initialized by solving the multiple regression problem with $\log\left(\frac{\underline{N}}{N}\right)=X\beta +\epsilon$, where ϵ is a normally distributed error vector with nine independent components. Goodness of fit was assessed by Pearson Χ^2^. The only thing to be changed for simplified models is the X matrix by removing the appropriate column(s) to eliminate a parameter or parameters not in a simplified model. The results for fitting the models with IRLS [29] computed with relative error <10^−8^ after nine iterations of IRLS are summarized in the table below.

The full epistatic model with three genes fitted the ratios from three crosses (Table 7). The only epistatic interaction that could possibly be dropped was between the A (WT) and B (Wrap) genes (αβ). This raised the question of whether or not any one of the additive allelic effects could be dropped altogether. This turned out to be the β allelic effect (for Wrap). A model without the B gene for Wrap did fit the progeny counts of the three crosses (Χ^2^ = 9.96, df = 5, *p* = 0.08). The conclusion was that at least two genes control conidiophore phenotype. An additive model and the model without gene effects was then used to assess the heritability. The heritability of H^2^ was typical for quantitative traits [26], namely, about 0.23.

The maximum likelihood estimates of parameters for the inheritance model are tabled (Table 8). The two promising models are fairly consistent across model parameters. One of these models has three loci, and the other has only two loci.

The allelic and epistatic effects are fairly stable between the two models. The C (Bulky) gene appears to have the largest effect both additively and epistatically with both B (Wrap) and A (WT). There also appears to be a strong epistatic interaction between B (Wrap) and C (Bulky) alleles.

### 3.6. Architectural Phenotype May Impact Colonization Capacity in N. crassa

**Figure 7:**
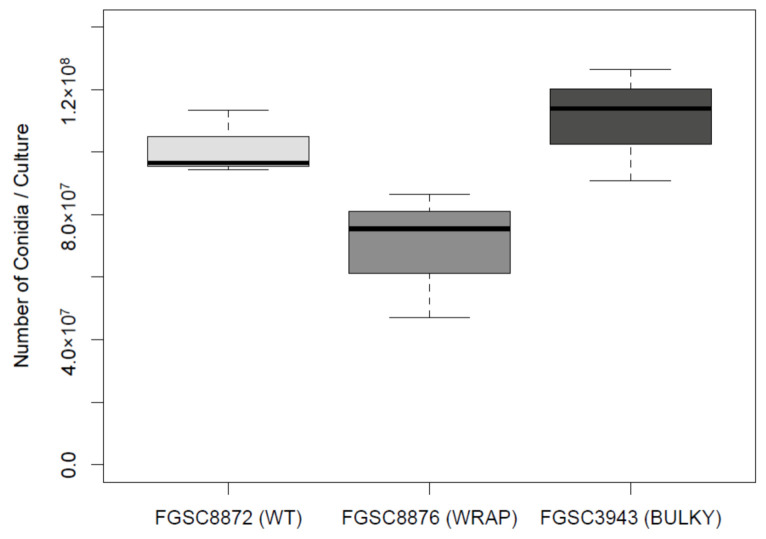
Number of conidia suspended in water from an 82 mm diameter nitrocellulose membrane by each conidiophore phenotype. The dataset includes three biological replicates. The box shows the 25th and 75th percentiles, with the median denoted as a line inside. The whiskers represent the range of data. The *t*-test results for pairs are as follows: WT–Wrap, *p* = 0.09541; Wrap–Bulky, *p* = 0.06079; WT–Bulky, *p* = 0.505.

**Figure 8:**
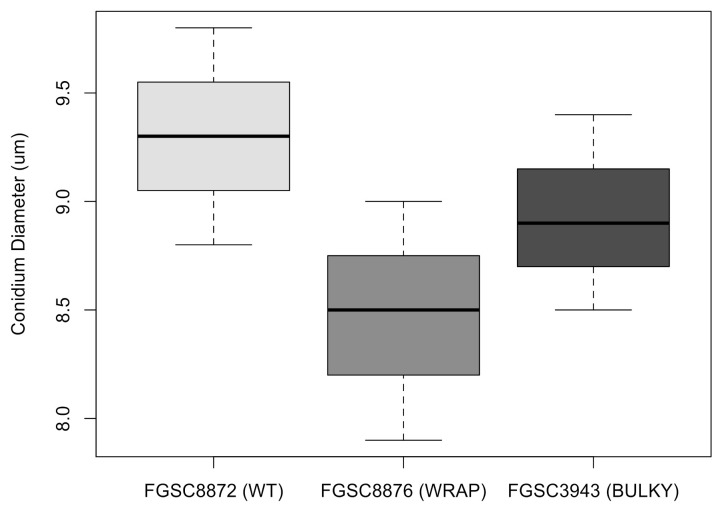
Average conidium diameter in microns when suspended in water for each architectural phenotype. The dataset includes three biological replicates. The box shows the 25th and 75th percentiles, with the median denoted as a line inside. The whiskers represent the range of data. The *t*-test results for pairs are as follows: WT–Wrap, *p* = 0.125; Wrap–Bulky, *p* = 0.3219; WT–Bulky, *p* = 0.3995.

Previous work conducted in *Aspergillus* has shown that conidiophore architecture impacts the colonization capability of the organism [8]. To investigate whether this may also be true in *N. crassa*, we quantified the average number of conidia produced by each conidiophore phenotype, using a representative strain for each. There was no statistically significant difference in the number of conidia produced by each phenotype when suspended in liquid culture (Figure 7). Additionally, we found no statistically significant difference in average conidium diameter produced by each phenotype (Figure 8).

**Figure 9:**
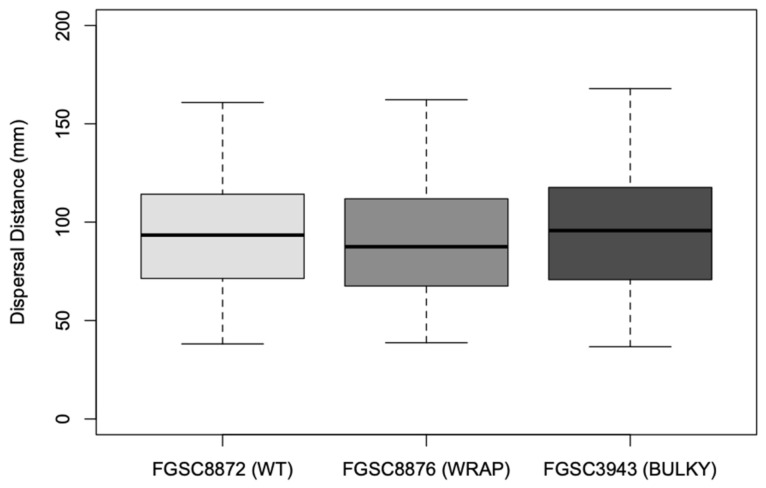
Distance in mm to the center of each colony on sorbose + fructose + glucose (SFG) medium from the center of each nitrocellulose membrane. The dataset includes two biological replicates. The box shows the 25th and 75th percentiles, with the median denoted as a line inside. The whiskers represent the range of data. The *t*-test results for pairs are as follows: WT–Wrap, *p* = 0.0587; Wrap–Bulky, *p* = 3.101 × 10^−5^; WT–Bulky, *p* = 0.3466.

**Figure 10:**
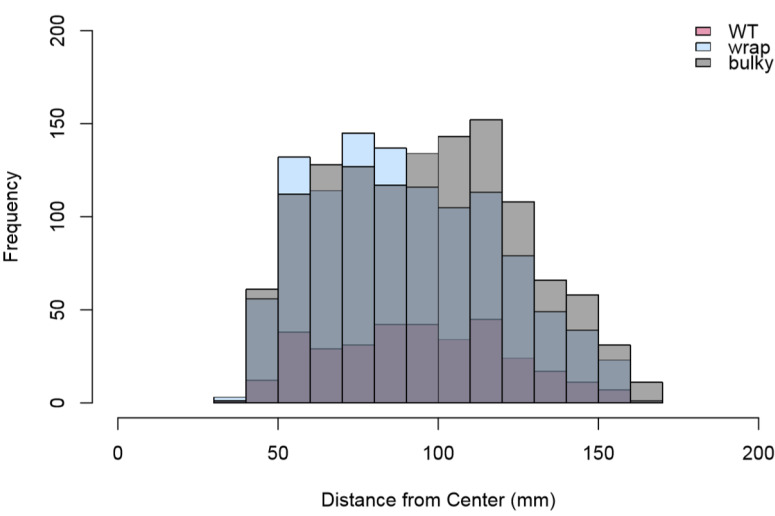
Histogram of distances in mm to the center of each colony from the center of each nitrocellulose membrane. Total colony counts combining two replicates are as follows: WT = 334, Wrap = 1113, Bulky = 1252. Results of two-sample Kolmogorov–Smirnov tests for each pair are as follows: WT–Wrap D = 0.090973, *p* = 0.02849; Wrap–Bulky D = 0.09624, *p* = 3.702 × 10^−5^; WT–Bulky D = 0.055465, *p* = 0.3922.

We also explored sporulation behavior on SFG medium, where mature conidiophores were placed at the center of a plate to allow for sporulation and subsequent colonial growth. Interestingly, fewer WT colonies (*n* = 334) developed compared to Wrap and Bulky (*n* = 1113 and *n* = 1252, respectively), suggesting a difference in sporulation behavior in an aerial versus aqueous environment. We quantified the distance from the center of each nitrocellulose membrane to the center of each colony. The distribution of these distances are referred to as the spore shadow. Two replicate datasets were combined, as there was no significant difference between replicates of a phenotype following a two-sample Kolmogorov–Smirnov test [30]. Sporulation by Wrap conidiophores resulted in colonies at a significantly closer distance when compared to that of Bulky colonies (*p* = 3.101 × 10^−5^) (Figure 9). No significant sporulation distance difference was found for the other pair combinations; however, differences in the distribution of colony distances were found between pairs WT and Wrap (D = 0.090973, *p* = 0.02849) and Wrap and Bulky (D = 0.09624, *p* = 3.702 × 10^−5^) by two-sample Kolmogorov–Smirnov tests (Figure 10).

## 4. Discussion

Conidiophore development in *N. crassa* has been thoroughly documented over decades of study [31]. Genetic, temporal, and environmental signals guiding this process are well understood [4,31]; however, little is known about conidiophore morphological variation, particularly in natural populations. The *N. crassa* collection of Louisiana isolates provides a convenient tool for characterizing variation in a natural population and has previously been used to describe local adaptations and reveal novel gene functions [11,12,13]. Following their collection from nature, these strains underwent conidial plating to remove possible fungal contaminants and were maintained vegetatively at the FGSC [32]. It is entirely likely that these isolates are heterokaryotic, reflective of the genetic variation observed in the wild. By using these populations as is, we aimed to capture the full spectrum of natural variation in conidiophore architecture.

By utilizing this population set of 21 wild strains to explore morphological variation in the conidiophore, we identified three novel architectural phenotypes: Wild Type, Bulky, and Wrap. These three phenotypes are consistently displayed throughout conidiophore development and are likely not the result of temporal disparity along the same growth trajectory. Interestingly, no clear dependence of phenotype on collection location or substrate was observed, suggesting a genetic component to this variation. To further explore this, we conducted crosses between representative strains for each phenotype. By quantifying the subsequent progeny conidiophores of these crosses, we were able to fit a model for inheritance. Our resulting model suggests that at least two genes control conidiophore architectural phenotype with an estimated heritability of 0.23.

Future work should seek to identify these genetic variants and characterize transcriptional profiles for conidiophores of different architectural phenotypes. Many key genetic players of conidiophore development have already been identified and thoroughly characterized, most notably the *con* genes [33], and transcriptomic data have elucidated gene expression profiles throughout wild-type conidiophore development [7]. Interestingly, the Bulky conidiophore phenotype resembles that of the *attenuated* (*at*) knockout mutant, where conidia form in dense aggregates [34]. However, we do not observe slowed growth and pigmentation in the Bulky wild isolates as is seen in *at* mutants. Identifying and characterizing additional genes possibly underlying WT, Bulky, and Wrap phenotypes would provide greater understanding of conidiophore development through a morphological lens and perhaps lend insight into the natural population structure of these wild isolates.

To investigate a potential environmental impact of conidiophore architecture, we evaluated the effect of conidiophore phenotype on sporulation in both an aqueous suspension and aerial environment. No significant effect was detected on the number of conidia or average conidium diameter when suspended in water, nor was there any difference in wettability of conidia while washing off a plate. This indicates that the conidiophore architectural phenotype is not due to difference in hydrophobicity of the conidia. In an aerial environment, fewer conidia from Wrap conidiophores germinated compared to Bulky. Additionally, spore shadows by conidia from Wrap conidiophores fit a different distribution compared to that of both WT and Bulky groups. This suggests that conidiophore architectural phenotype may impact colonization capacity of the organism. Sporulation experiments should be conducted at a larger scale to more effectively determine the maximum dispersal distance of each conidiophore phenotype and, hence, effective population size and neighborhood size [35,36]. Furthermore, it is possible that conidiophore phenotype may contribute to microspatial variation through altering effective population size and neighborhood size, as some strains exhibiting different architectural phenotypes were collected from the same substrate in the same location [37].

It remains a challenge to relate complex traits describing growth and form to their underlying genes [1]. Doing so requires genomics [13,38], transcriptomics [12], and/or metabolomics [39], as well as high-throughput approaches to phenotyping. Morphological diversity presents an attractive opportunity for image analyses and phenotyping at a large scale, as shown in other model fungal systems such as yeast [40,41]. Phenomics tools like Digital Imaging of Root Traits (DIRT) have been developed specifically to quantify architectural features in plant root systems [42]. Although filamentous fungi bear some similarities to roots, the unique morphology of conidiophores is best characterized with a tool specifically designed for their structure. We presented a novel method to classify brightfield images of conidiophores into three architectural phenotypes and extract important features for their classification. This same approach can be applied to characterize conidiophores of other filamentous fungi and may even be used in conjunction with DIRT to characterize morphology in the symbiotic relationship between roots and arbuscular mycorrhiza [43,44]. To further improve our method, automation in sample collections and blurred region removal could be implemented. Performance in model training was also limited by the sample size in our study and domain-specific features.

## Figures and Tables

**Table 1:** Classifications of conidiophores belonging to each wild isolate (chi-squared test for independence: Χ^2^ = 380.92, df = 40, *p* < 2.2 × 10^−16^). Individual conidiophore counts vary with each strain. Phenotypes are balanced for classifier training described below.

Strain	WT	Bulky	Wrap	Total
FGSC0847	22	0	4	26
FGSC2221	26	1	3	30
FGSC2222	13	0	8	21
FGSC2223	10	2	3	15
FGSC2224	16	23	6	45
FGSC2228	39	0	5	44
FGSC2229	14	54	48	116
FGSC2489	32	7	1	40
FGSC3199	17	0	2	19
FGSC3200	30	5	23	58
FGSC3211	20	16	7	43
FGSC3212	54	2	6	62
FGSC3943	32	80	52	164
FGSC8870	19	2	9	30
FGSC8871	16	6	17	39
FGSC8872	14	0	4	18
FGSC8873	8	11	13	32
FGSC8874	20	3	14	37
FGSC8876	15	21	42	78
FGSC8878	20	26	12	58
FGSC8879	6	12	11	29
Total	443	271	290	

**Table 2:** Performance of ResNet-50 classification on training, validation, and test sets. Accuracy, precision, and recall are used for evolution.

	Accuracy	Precision	Recall
Training	0.9632	0.9644	0.9632
Validation	0.7879	0.7890	0.7879
Test	0.7576	0.7540	0.7576
External test set	0.6779	0.6802	0.6639

**Table 3:** Confusion table for the test set. Each row indicates different true labels, and each column indicates different predictions. In the test set, the accuracies are 0.9545 (Bulky), 0.5909 (Wrap), and 0.7273 (WT).

	Bulky	Wrap	WT
Bulky	21	1	0
Wrap	4	13	5
WT	2	4	16

**Table 4:** The cell probabilities for the fully epistatic inheritance model with three genes in three crosses. The probability of phenotype j from cross i is K_ij_.

	A (WT)	B (Wrap)	C (Bulky)
A × B	$K_{11}=e^{\lambda }e^{\alpha }e^{-\beta }e^{\alpha \beta }$	$K_{12}=e^{\lambda }e^{-\alpha }e^{\beta }e^{\alpha \beta }$	$K_{13}=e^{\lambda }e^{-\alpha }e^{-\beta }e^{-\alpha \beta }$
B × C	$K_{21}=e^{\lambda }e^{-\beta }e^{-\gamma }e^{-\beta \gamma }$	$K_{22}=e^{\lambda }e^{\beta }e^{-\gamma }e^{\beta \gamma }$	$K_{23}=e^{\lambda }e^{-\beta }e^{\gamma }e^{\beta \gamma }$
A × C	$K_{31}=e^{\lambda }e^{\alpha }e^{-\gamma }e^{\alpha \gamma }$	$K_{32}=e^{\lambda }e^{-\alpha }e^{-\gamma }e^{-\alpha \gamma }$	$K_{33}=e^{\lambda }e^{-\alpha }e^{\gamma }e^{\alpha \gamma }$

**Table 5:** Multinomial counts of progeny phenotypes from each of the three crosses.

	A (WT)	B (Wrap)	C (Bulky)
A × B	$N_{11}=156$	$N_{12}$= 205	$N_{13}=253$
B × C	$N_{21}=94$	$N_{22}$= 155	$N_{23}$= 275
A × C	$N_{31}=202$	$N_{32}$= 230	$N_{33}$= 198

**Table 6:** The derivatives of the model cell probabilities are stored in the below 9 × 7 derivative matrix X. Each row captures the parameters present in an expected cell probability K_ij_ of one of the three phenotypes j in cross i (Table 4). The X matrix captures the structure of the model. Dropping/adding columns corresponds to dropping/adding parameters in a model.

Parameters/K	λ	α	β	γ	αβ	βγ	αγ
$K_{11}$	1	1	−1	0	1	0	0
$K_{12}$	1	−1	1	0	1	0	0
$K_{13}$	1	−1	−1	0	−1	0	0
$K_{21}$	1	0	−1	−1	0	−1	0
$K_{22}$	1	0	1	−1	0	1	0
$K_{23}$	1	0	−1	1	0	−1	0
$K_{31}$	1	1	0	−1	0	0	1
$K_{32}$	1	−1	0	−1	0	0	−1
$K_{33}$	1	−1	0	1	0	0	1

**Table 7:** A nested hierarchy of inheritance models was successfully fitted with at least two genes controlling the conidiophore architectural phenotype to the counts of progeny phenotypes from three crosses (Table 5). Nine iterations were necessary to achieve the desired error tolerance of 10^−8^ with iteratively reweighted least square (IRLS). Recommended models are bolded along with their goodness of fit to the counts of phenotypes in crosses. A null hypothesis (H0) is tested against an alternative (HA) with the chi-squared test having degrees of freedom (df).

Model	Χ^2^	df	*p*	X^2^_HA_ − X^2^_H0_	df	*p* for HA vs. H0	Notes
**Full epistatic**	0.98	2	**0.61**	-	-	-	H0 = full epistatic
αβ=0	8.39	3	0.04	8.39 − 0.98 = 7.41	1	0.004	H0 = full epistatic
αβ=α=0	25.31	5	0.001	25.31 − 0.98 = 24.33	2	<0.00001	H0 = full epistatic
αβ=β=0	9.96	5	**0.08**	9.96 − 0.98 = 8.98	2	0.01	H0 = full epistatic
βγ=0	9.32	3	0.02	9.32 − 0.98 = 8.34	1	0.004	H0 = full epistatic
αγ=0	84.57	3	<0.0001	84.57 − 0.98 = 83.59	1	<0.00001	H0 = full epistatic
αβ=βγ=αγ=0 additive	95.76	4	<0.0001	95.76 − 0.98 = 94.78	3	<0.00001	H0 = full epistatic
environmental	124.46	8	<0.0001	124.46 − 0.98 = 123.48	7	<0.00001	H0 = full epistatic
heritability							H^2^ = (124.46 − 95.76)/124.46 = 0.23 H0 = environmental model H1 = full additive model

**Table 8:** Maximum likelihood estimates of allelic effects and epistatic effects in a two or three locus model of inheritance. The full epistatic model with three genes has three allelic effects and three epistatic interactions. The two-gene model with gene B (Wrap) removed has two allelic effects for A (WT) and C (Bulky) and two epistatic interactions between A (WT) and C (Bulky) and between B (Wrap) and C (Bulky). The standard errors were obtained from the square roots of the diagonal elements of the inverse of the information matrix N$X^{\prime }AX$.

Parameters	Full Epistatic 3 Genes	αβ=β=0 2 Genes
λ	5.32 ± 0.0040	5.32 ± 0.0030
α	−0.08 ± 0.0048	0.02 ± 0.0048
β	0.05 ± 0.0061	0
γ	0.53 ± 0.0047	0.53 ± 0.0044
αβ	−0.15 ± 0.0060	0
βγ	0.20 ± 0.0067	0.23 ± 0.0061
αγ	−0.60 ± 0.0064	−0.58 ± 0.0062

## Appendix A

**Table A1:** Population of wild Louisiana isolates of *N. crassa*. Strains used in this study were obtained from the FGSC. Mating type (Mat) of each strain is denoted by A or a.

Strain Number	FGSC	Perkins	Mat	Strain Provenance	Collection Site	Substrate/Annotation
**Wild Strains**						
D110	8870	4448	A	Dettman, J.	Franklin, LA	sugarcane
D111	8871	4449	a	Dettman, J.	Franklin, LA	sugarcane
D112	8872	4453	A	Dettman, J.	Franklin, LA	sugarcane
D114	8874	4464	A	Dettman, J.	Franklin, LA	sugarcane
D116	8876	4481	a	Dettman, J.	Franklin, LA	sugarcane
D118	8878	4491	a	Dettman, J.	Franklin, LA	sugarcane
JW09	2229		A	Welch, J.	Welsh, LA	burned grass
JW10	2229		A	Welch, J.	Welsh, LA	burned grass
JW59	3200		a	Welch, J.	Coon, LA	burned stumps
JW66	3211		a	Welch, J.	Sugartown, LA	Pine burn
JW70	3199		A	Welch, J.	Coon, LA	burned stumps
JW75	3943		a	Welch, J.	Houma, LA	sugarcane burn
	847		A	Lein	Louisiana	sugarcane burn
D113	8873	4454	a	Dettman, J.	Franklin, LA	sugarcane
D119	8879	4500	a	Dettman, J.	Franklin, LA	sugarcane
JW20	3212		A	Welch, J.	Ravenswood, LA	bonfire
JW76	3943		a	Welch, J.	Houma, LA	sugarcane burn
JW159	2221		a	Welch, J.	Houma, LA	sugarcane burn
JW160	2222		A	Welch, J.	Iowa, LA	grass burn
JW162	2223		a	Welch, J.	Iowa, LA	grass burn
JW164	2224		a	Welch, J.	Marrero, LA	wood burn
JW167	2228		a	Welch, J.	Roanoke, LA	grass burn
OR74A	2489		A	FGSC	Marrero, LA	unknown
